# *Yamatochaitophorus yichunensis*, a new species of aphid (Aphididae: Chaitophorinae) from northeast China

**DOI:** 10.3897/zookeys.612.7873

**Published:** 2016-08-23

**Authors:** Li-Yun Jiang, Jing Chen, Ge-Xia Qiao

**Affiliations:** 1 Key Laboratory of Zoological Systematics and Evolution, Institute of Zoology, Chinese Academy of Sciences, No. 1–5 Beichen West Road, Chaoyang District, Beijing 100101, P.R. China

**Keywords:** Aphididae, Chaitophorinae, China, new record, new species, Yamatochaitophorus

## Abstract

*Yamatochaitophorus yichunensis* **sp. n.** is described from specimens collected in northeast China on *Acer tegmentosum* (Aceraceae). *Yamatochaitophorus* is also a new generic record for China. Type specimens are deposited in the National Zoological Museum of China, Institute of Zoology, Chinese Academy of Sciences, Beijing, China (NZMC) and the Natural History Museum, London, UK (BMNH).

## Introduction

The genus *Yamatochaitophorus* was erected by Higuchi (1972) for *Trichaitophorus albus* Takahashi, 1961, which was described from an unidentified *Acer* sp. in Japan. The alata and embryo of *Yamatochaitophorus albus* were described by Chakrabarti and Mandal (1986) from several *Acer* spp. in India. The genus has until now been monotypic (Favret 2016). Here, a second species, *Yamatochaitophorus yichunensis* sp. n. is described based on the specimens collected in northeast China on *Acer tegmentosum* (Aceraceae); the genus is newly recorded in China.

In the Chaitophorinae, three genera are associated with the plant family Aceraceae; *Periphyllus* van der Hoeven, *Trichaitophorus* Takahashi and *Yamatochaitophorus* Higuchi. *Yamatochaitophorus* is closely related to *Trichaitophorus*, also distributed in Eastern Asia (China, Japan and India), but can be distinguished from *Trichaitophorus* by the larger number of marginal setae and fewer eye facets, as well as by the shape and length of the dorsal setae (Higuchi 1972; Chakrabarti and Mandal 1986). *Yamatochaitophorus* is similar to *Periphyllus* on the same host plants; cauda always rounded, anal plate entire, siphunculi with reticulations in alatae etc. However, it may be distinguished from *Periphyllus* by the following characters: eyes with approx. 25 facets (the latter: many eye facets), antennae 5-segmented in apterae (the latter: 6-segmented); first tarsal segments with 3 ventral setae (the latter: first tarsal segments with 5–7 ventral setae); body small, elongate oval, less than 1.36mm in apterae (the latter: body relatively large, mostly elliptical); without “aestivales” form in summer (the latter: with “aestivales” form, modified first-instar larvae of resting stage) (Higuchi 1972; Richards 1972; Junkiert et al. 2011).

## Materials and methods

Reliable aphid taxonomy requires slide-mounted specimens that are undistorted, but with the body contents fully cleared to make it possible to see surface details clearly. Specimens were placed in a 10% solution of potassium hydroxide and heated for 10–20 minutes or until body contents have softened. They were taken successively through distilled water, 70% EtOH and 95% EtOH, then transferred to clove oil for a minimum of 5 minutes, and finally mounted in Canada balsam. The descriptions and drawings provided here were produced from slide-mounted specimens using a Leica DM4000B and drawing tube. The photomicrograph images were prepared with a Leica DM2500 using DIC illumination, and processed with Automontage and Photoshop software.

Specimens of *Yamatochaitophorus albus* from Japan and India were obtained on loan from the Natural History Museum, London, UK, (BMNH) for comparison with our material.

Aphid terminology in this paper generally follows that of Higuchi (1972) and Chakrabarti and Mandal (1986). The units of measurement in this paper are millimetres (mm). The holotype and some paratypes are deposited in the National Zoological Museum of China, Institute of Zoology, Chinese Academy of Sciences, Beijing, China (NZMC); the other paratypes are deposited in the Natural History Museum, London, UK (BMNH).

## Taxonomy

### Yamatochaitophorus

Taxon classificationAnimaliaHemipteraAphididae

Higuchi, 1972

Yamatochaitophorus Higuchi: Chakrabarti and Mandal 1986: 334; Remaudière and Remaudière 1997: 168; Blackman and Eastop 1994: 925.

#### Type-species.

*Trichaitophorus albus* Takahashi, 1961; by original designation.

#### Generic diagnosis.

Apterae: Head fused with pronotum. Eyes with approx. 25 facets or less. Antenna 5-segmented, occasionally 6-segmented, shorter than body. Antennal segments without secondary rhinaria. Ultimate rostral segment with 0–2 accessory setae. Dorsal body covered with O- or C-like tubercles, and dorsal setae of body long, thick, flattened with knobbed or blunt apices; ventral body with spinulose stripes. First tarsal segments with three setae. Abdominal tergites I-VII solidly fused, each with paired spinal and marginal setae. Siphunculi short, truncate, without reticulations. Cauda rounded, anal plate broadly rounded, genital plate transverse elliptical. In alatae (Chakrabarti and Mandal 1986): antenna 6-segmented, segment III with moderately protuberant rhinaria. Dorsal setae long and acute. Siphunculi with reticulation.

### Yamatochaitophorus albus

Taxon classificationAnimaliaHemipteraAphididae

(Takahashi, 1961)

Table 1

Trichaitophorus albus Takahashi, 1961: 8.

#### Specimens examined.

1 apterous viviparous female, Japan: Suganuma (Tumma Pref.), 22 July 1967, on *Acer* sp., coll. H. Higuchi, leg. H. Higuchi (BMNH); 1 apterous viviparous female, India: Bhuinder (U.P.), 30 May 1980, on *Acer acuminatum*, coll. S. Saha, leg. S. Chakrabarti (BMNH) (Morphometric data of the specimen is in the table1).

#### Distribution.

Japan, India, Siberia.

#### Host plants.

*Acer acuminatum*, *Acer mono*, *Acer ukurunduense*, *Acer villosum* and *Acer* sp.

### Yamatochaitophorus yichunensis sp. n.

Taxon classificationAnimaliaHemipteraAphididae

http://zoobank.org/4B15E539-219C-4529-A698-5FE963CC56CE

Figures 1–14 , 15–27 , 28–30 , Table 1

#### Specimens examined.

Holotype: apterous viviparous female, China: Heilongjiang Province, Yichun City (Tangwanghe National Forest Garden, E 129.54°, N48.45°, Alt. 360 m), 21 July 2015, No. 35896-1-1-1, on *Acer tegmentosum*, coll. G.X. Qiao.

*Paratypes*: 8 apterous viviparous females, with the same collection data as holotype (NZMC); 2 apterous viviparous females, with the same collection data as holotype (BMNH).

#### Etymology.

The specific name *yichunensis* is based on the type locality of the species.

#### Diagnosis.

Body small, elongate oval, adults pale yellow in life. Dorsal body covered with O- or C-like tubercles. Antenna 5-segmented, half as long as body or shorter, with processus terminalis longer than the base of last segment. Ultimate rostral segment with 1 or 2 accessory setae. Embryo with long, thick and acute spinal setae similar to marginal setae.

#### Description.

*Apterous viviparous female*: Body elongate oval, pale yellow in life (Figs 28–30). **Mounted specimens.** Whole dorsum pale (Fig. 15). For morphometric data see Table 1. Dorsum with longitudinal spinal ridge, and covered with “O-”or “C-” like tubercles, ventral marginal area with spinulose stripes (Figs 1, 6–9, 15, 20). Dorsal setae of body thick, long or short, flattened with knobbed or blunt apices, with well-developed tubercles at bases (Figs 1, 4–9, 20–22); ventral setae very sparse, very short and fine-pointed 1/4-1/3 of length of dorsal setae.

**Figures 1–14.**
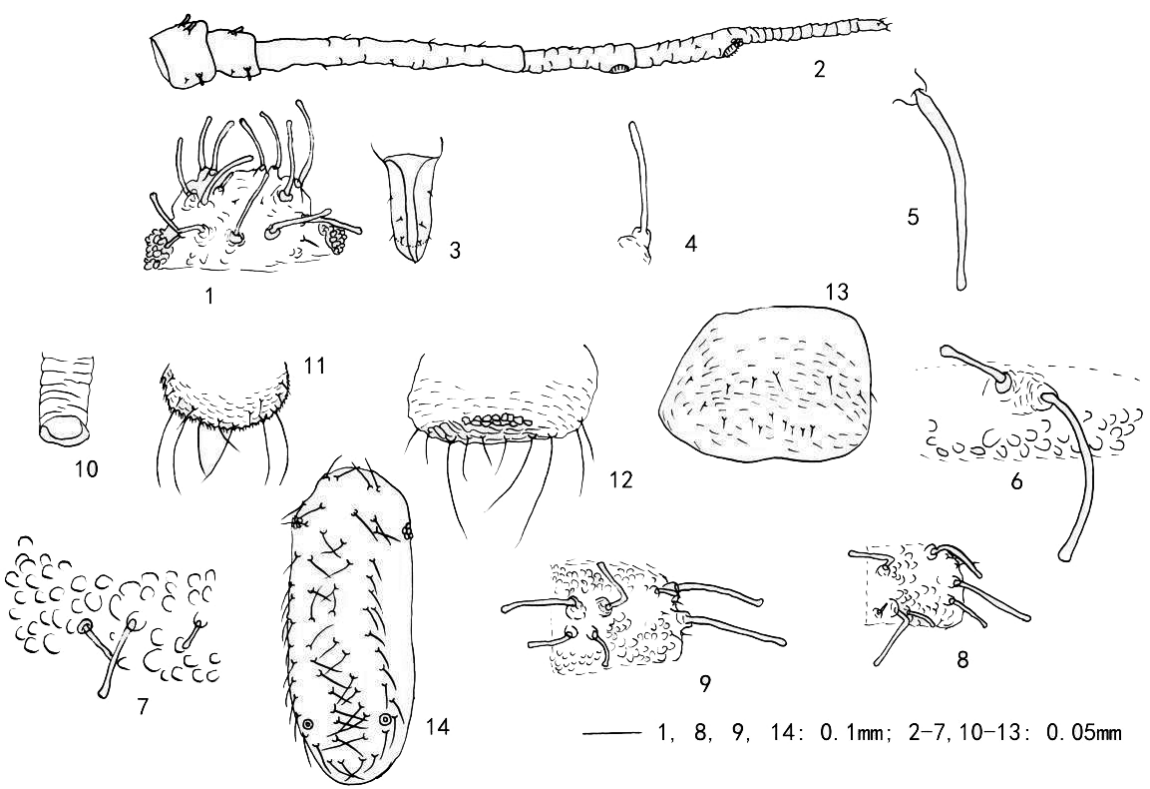
*Yamatochaitophorus yichunensis* sp. n. Apterous viviparous female: **1** dorsal view of head **2** antennal segments I–V **3** ultimate rostral segment **4** cephalic seta **5** marginal seta on abdominal tergite I **6** spinal setae and tubercles on abdominal tergite I in right, setal tubercles at base shown **7** tubercles on pleuro-marginal area on abdominal tergite IV **8** dorsal view of pronotum in right, dorsal setae, setal tubercles at base and tubercles shown **9** dorsal view of mesonotum in right, dorsal setae, setal tubercles at base and tubercles shown **10** siphunculi **11** cauda **12** anal plate **13** genital plate **14** embryo, dorsal setae shown.

**Figures 15–27.**
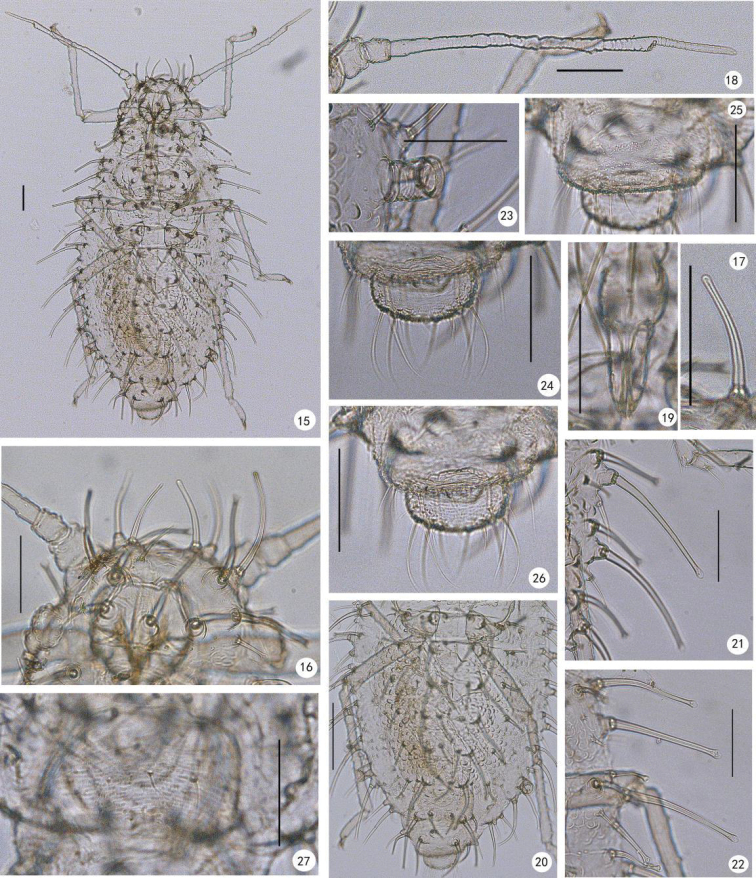
*Yamatochaitophorus yichunensis* sp. n. Apterous viviparous female: **15** dorsal view of body **16** dorsal view of head, dorsal setae shown **17** cephalic seta **18** antenna **19** ultimate rostral segment **20** C- or O-like tubercles on abdominal tergites, **21** marginal setae on abdominal tergites III–IV **22** marginal setae on meso- and metanotum **23** siphunculus **24** cauda **25** anal plate **26** cauda and anal plate, mosaic-like ornamentation on distal ventral area of anal plate shown **27** genital plate. Scale bars: 0.10 mm.

**Figures 28–30.**
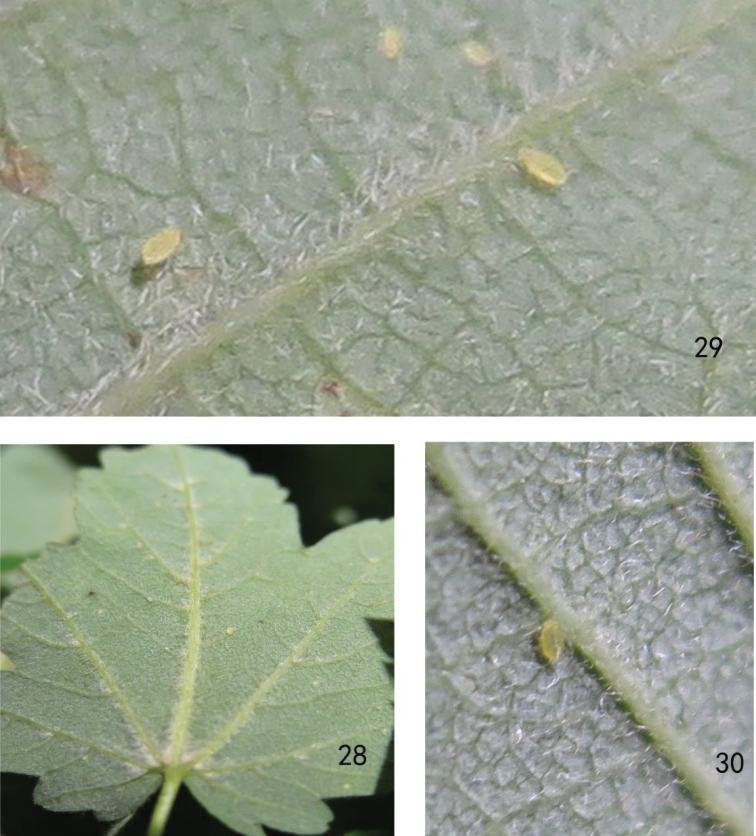
*Yamatochaitophorus yichunensis* sp. n. Apterous viviparous female: **28, 29** population colonising the underside of leaves **30** living aphid.

**Table 1.** Morphometric data for apterous viviparous females of *Yamatochaitophorus yichunensis* sp. n. (n = 11, with means in brackets) and *Yamatochaitophorus albus* (Takahashi) (reliable morphometric data was only obtainable from one specimen, from India), the measurement is in mm.

	*Yamatochaitophorus yichunensis* sp. n.	*Yamatochaitophorus albus* (Takahashi)
Characters	Apterous viviparous females (n=11)	Apterous viviparous female (n=1)
Body length	0.800–1.360 (0.928)	1.300
Body width	0.310–0.590 (0.391)	0.620
Antenna	0.328–0.602 (0.396)	0.505
Antennal segment I	0.035–0.050 (0.040)	0.055
Antennal segment II	0.029–0.038 (0.032)	0.030
Antennal segment III	0.090–0.224 (0.127)	0.104
Antennal segment IV	0.050–0.095 (0.060)	0.060
Antennal segment V	–	0.090
Base of antennal segment V (VI)	0.055–0.075 (0.065)	0.075
Processus terminalis	0.085–0.119 (0.080)	0.092
Ultimate rostral segment	0.055–0.085 (0.065)	0.080
Hind femur	0.124–0.224 (0.156)	0.214
Hind tibia	0.214–0.383 (0.268)	0.348
Second hind tarsal segment	0.075–0.100 (0.084)	0.100
Siphunculus	0.015–0.050 (0.026)	0.050
Basal width of siphunculus	0.020–0.040 (0.029)	0.065
Distal width of siphunculus	0.015–0.040 (0.026)	0.045
Cauda	0.040–0.050 (0.045)	0.055
Basal width of cauda	0.060–0.090 (0.081)	0.095
Basal diameter of antennal segment III	0.015–0.025 (0.019)	0.020
Width of hind tibia at mid length	0.020–0.025 (0.023)	0.035
Longest dorsal cephalic seta	0.070–0.095 (0.077)	0.109
Longest marginal seta on abdominal tergite I	0.159–0.199 (0.170)	0.184
Longest seta on abdominal tergite VIII	0.104–0.124 (0.111)	0.174
Longest seta on antennal segment III	0.003–0.005 (0.004)	0.005
Longest seta on hind tibia	0.020–0.040 (0.028)	0.070

*Head*. Frons convex, antennal tubercles poorly developed (Figs 1, 16); dorsum of head with short wrinkles (Figs 1, 16). Body dorsal setae long thick, flat at apices, with well-developed tubercles at bases (Fig. 4). Head with 4–5 long thick and 2–3 short dorsal setae between antennae, and two pairs of long thick and 0–2 short dorsal setae between eyes (Figs 1, 16). Eyes with approx. 25 facets. Antennae 5-segmented (Figs 2, 18), 0.41–0.50 times as long as body; length in proportion of segments : 22–42, 18–33, 100, 41–59, 33–67+48–74, respectively, processus terminalis 1.07–1.60 times as long as the base of the segment. Segments III- V weakly imbricated. Antennal setae very short and blunt, setae on inside of segments I-II short, thick and flattened at apices; segments I–V with 4, 3, 1–5, 1, 1+0 setae, respectively; apex of processus terminalis with 2–4 setae. Longest setae on segment III 0.13–0.25 times as long as basal diameter of the segment. Primary rhinaria ciliated, secondary rhinaria absent (Figs 2, 18). Rostrum (Figs 3, 19) reaching mid-coxae; ultimate rostral segment wedge-shaped, 1.57–2.00 times as long as its basal width, 0.72–0.85 times as long as second hind tarsal segment, with 1 or 2 accessory setae.

*Thorax* (Fig. 15). Pronotum with 1 pair of long thick anterior spinal setae, 1–3 pairs of posterior spinal setae (of which 1 pair is long and thick) and 3–5 pairs of marginal setae (of which two pairs are long and thick) (Fig. 8); mesonotum with 3–5 pairs of spino-pleural setae (of which two pairs are long and thick) and 2–5 pairs of marginal setae (of which two pairs are long and thick) (Figs 9, 22); metanotum with 4–8 spino-pleural (of which two pairs are long and thick) and 2–5 pairs of marginal setae (of which two pairs are long and thick) (Fig. 22). Legs normal. Hind femur 0.96–1.46 times as long as antennal segment III. Hind tibia 0.26–0.32 times as long as body. Setae on legs long and pointed, length of setae on hind tibiae 1.00–1.60 times as long as middle diameter of the segment. First tarsal chaetotaxy: 3, 3, 3.

*Abdomen*. Abdominal tergites I-VII each with one pair of spinal and one pair of marginal long thick setae with flattened or expanded apices (the expanded part is membranous) (Figs 15, 21). In addition, abdominal tergite I with 3–10 spino-pleural and 1–5 pairs of marginal shorter setae (Figs 5–6, 20); tergite II with 7–8 spino-pleural and 2–3 pairs of marginal setae; tergite III with 3–8 spino-pleural and 1–5 pairs of marginal shorter setae (Fig. 21); tergites IV-V each with 3–10 spino-pleural and 2–4 pairs of marginal shorter setae (Figs 7, 21), respectively; tergite VI with 3–10 spino-pleural and 2–4 pairs of marginal shorter setae; tergite VII with 3–6 spino-pleural and 2–3 pairs of marginal shorter setae; tergite VIII with 6–8 setae (Fig. 15). Length of longest marginal setae on tergite I 6.60–11.00 times as long as basal diameter of antennal segment III; dorsal setae on tergite VIII 4.60–7.00 times as long as basal diameter of antennal segment III. Spiracles oval, opened or closed; spiracular plates large, oval or round. Siphunculi (Figs 10, 23) short truncated, with weak transverse imbrications, without polygonal reticulation, flanges developed, 0.67–1.33 times as long as its basal width, about 0.40–1.00 time as long as cauda. Cauda (Figs 11, 24, 26) short, rounded, slightly constricted at base, with spinulose imbrications, 0.50–0.67 times as long as basal width, with 8–14 setae, among 6 long thick setae. Anal plate (Figs 12, 25, 26) broadly rounded, with spinulose short lines; with 15–20 setae, including two long thick setae; and distal ventral area of anal plate with mosaic-like ornamentation (Fig. 26). Genital plate (Figs 13, 27) transverse oval, with spinulose transverse lines; with two pairs of anterior setae, and 9–13 posterior setae. Four gonapophyses.

*Embryo* (Fig. 14): Dorsal setae of body long thick and acute. Head with two pairs of frontal setae and two pairs of mid-dorsal setae. Pro-, meso- and metanotum each with a single pair of spinal setae and two pairs of marginal setae. Abdominal tergites I-VII each with one pair of spinal and one pair of marginal setae; tergite VIII with two pairs of fine and short dorsal setae. Siphunculi short, truncated. Eyes with 6–7 facets.

#### Host plant.

*Acer tegmentosum* (Aceraceae). The species infested the underside of leaves of the host plant, and population density was low, with less than 20 individuals dispersed on the underside of a leaf (Figs 28, 29).

## Key to apterous viviparous females

**Table:** 

1	Abdominal tergites I-VII each with 3–10 spino-pleural (one occasionally), and 1–5 pairs of marginal shorter setae with flattened apices, besides 1 pair of spinal and 1 pair of marginal long and thick setae; ultimate rostral segment with 1 or 2 accessory setae; in embryos, dorsal body with long, thick and acute spinal setae, similar to marginal setae in shape	***Yamatochaitophorus yichunensis* sp. n.**
–	Abdominal tergites I-VII each with 1 or 2 spino-pleural and 2 marginal shorter setae with flattened apices, besides 1 pair of spinal and 1 pair of marginal long and thick setae; ultimate rostral segment without accessory setae; in embryos, the marginal setae are long, thick and acute, but the spinal setae are very short or indiscernible	***Yamatochaitophorus albus* (Takahashi)**

## Supplementary Material

XML Treatment for Yamatochaitophorus

XML Treatment for Yamatochaitophorus albus

XML Treatment for Yamatochaitophorus yichunensis
